# Experimental data of the study on H-rotor with semi-elliptic shaped bladed vertical axis wind turbine

**DOI:** 10.1016/j.dib.2018.06.063

**Published:** 2018-06-26

**Authors:** T. Micha Premkumar, S. Seralathan, R. Gopalakrishnan, T. Mohan, V. Hariram

**Affiliations:** School of Mechanical Sciences, Department of Mechanical Engineering Hindustan Institute of Technology and Science, Chennai, Tamil Nadu, India

**Keywords:** Vertical axis wind turbine, H-rotor, Semi-elliptic shaped blade, Performance, Low wind speed regimes

## Abstract

The performance and load test data of the proposed H-rotor with semi-elliptical shaped blade vertical axis wind turbine is carried out at the laboratory using 1 m diameter axial fan. India has a long coastline and low-wind velocity ranging from 3 to 10 m/s which is available everywhere in the country irrespective of climatic conditions. The data article is carried out at different aspect ratios along with tilt of the blades and without tilting of the blades. These data sets provide the researchers to further study experimentally as well as numerically in order to enhance the performance of the proposed VAWT. The data presented here are measured at wind velocity ranging from 3 to 6 m/s. The raw data captured using data acquisition system are processed and presented in a form so as to compare it with other typical VAWT.

**Specifications Table**

**Table:** 

Subject area	*Renewable energy*
More specific subject area	*Wind engineering*
Type of data	*Graphical figures and Tables*
How data was acquired	*Experimental setup* of H-rotor with semi-elliptic shaped blade by *using vane anemometer, torque sensor, RPM sensor along with data acquisition system and mechanical loading arrangement with a dynamometer*
Data format	*Raw, filtered, calculated, tabulated, analyzed, plotted*
Experimental factors	*Data are normalized as per the standards used in wind turbine studies*
Experimental features	H-rotor with semi-elliptic shaped bladed VAWT working based on the principle of drag is *tested at laboratory with wind speeds ranging from 3 m/s to 6 m/s*
Data source location	*Mechanical Engineering Department, Hindustan Institute of Technology and Science, Chennai,Tamilnadu, India*
Data accessibility	*Data on the performance study are included in this article*

**Value of the data**

• The highlight of this data set on H-rotor with semi-elliptic shaped bladed vertical axis wind turbine is to understand the design and aerodynamic behaviour of this type of wind rotor operating at low wind speed regimes.
• This data set would enable and assist the researchers in designing packaged installations of H-rotor with semi-elliptic shaped bladed vertical axis wind turbine for roof top power generation at urban homes.
• This data set would enable the people to develop a better aerodynamic design based on this measured data and it is also provides a benchmark for subsequent simulation studies.

## 1 Data

Several studies were performed on the blade shapes such as semi-circular, semi-elliptic, Benesh and Bach types to improve the power coefficient value [1], [2], [3], [4], [5] of Vertical Axis Wind Turbine (VAWT). Earlier, a new bucket shape effect of a non-conventional Savonius rotor named incurved Savonius rotor was proposed by Driss et al. [6]. Based on these earlier studies, it was found that blade shape plays a prominent role in improving the performance of vertical axis wind turbine. Traditionally, the self-starting capability of H-rotor is poor compared to Savonius style VAWT. In order to overcome this, it is intended in this data to incorporate the semi-elliptic shaped blades instead of conventional NACA air foil blades (Table 1).

**Table 1:** Specification details.

Shape of the blade = Semi-elliptic shaped
Aspect ratio (As = *H*/*D*) = 1.0–1.22
Projected diameter of the rotor (*D*) = 1 m to 0.820 m
Height of the rotor (*H*) = 1 m
No. of stage = Single stage
No. of blade = 3 nos.
Chord length of the blade = 0.2 m

The experimental investigation is carried out to obtain the data set for analyzing the performance of this non-conventional H-rotor with semi-elliptic shaped bladed vertical axis wind turbine by varying the aspect ratio (H/D) at low-wind speeds which is varied from 3 m/s to 6 m/s. The aspect ratio is varied from 1.0 to 1.22 in this data. The data is also obtained by tilting the blades by 15° and without tilting conditions. Table 2 in this data set shows the rotational speeds of the proposed VAWT with wind velocities. Table 3, Table 4 in this data set shows the variations of TSR and C_p_ for aspect ratio 1.22 and 1.0 respectively for blades with and without tilt at various load conditions.

**Table 2:** Wind velocity and the rotational speed of the proposed VAWT.

**Sl.No.**	**Wind velocity(m/s)**	**Vertical Axis Wind Turbine speed(RPM)**
1	3.08	6.2
2	3.21	7.1
3	4.56	11.2
4	4.75	12.6
5	5.26	15.8
6	6.31	22.1

**Table 3:** Variations of TSR and C_p_ for aspect ratio 1.22 for blades with and without tilt at various loads.

**Without any blade tilt**						**With blade tilt by 15°**	
**Load 100** **gms**		**Load 200** **gms**		**Load 300** **gms**		**Load 100** **gms**	
**Tip speed ratio (TSR)**	**Power coefficient *C***_**p**_	**Tip speed ratio (TSR)**	**Power coefficient *C***_**p**_	**Tip speed ratio (TSR)**	**Power coefficient *C***_**p**_	**Tip speed ratio (TSR)**	**Power coefficient *C***_**p**_
0.111248	0.010091	0.091716	0.014246	0.06362	0.009404	0.072589	0.01073
0.117119	0.012992	0.100729	0.014588	0.066797	0.012975	0.08764	0.012551
0.123126	0.013788	0.111804	0.015469	0.078354	0.017718	0.120541	0.01589
0.124773	0.016599	0.120803	0.015482				
		0.136817	0.015909				
		0.159504	0.018622				
		0.166695	0.020696

**Table 4:** Variations of TSR and *C*_p_ for aspect ratio 1 for blades with and without tilt at various loads.

**Without any blade tilt**						**With blade tilt by 15°**	
**Load 100** **gms**		**Load 200** **gms**		**Load 300** **gms**		**Load 100** **gms**	
**Tip speed ratio (TSR)**	**Power coefficient *C***_**p**_	**Tip speed ratio (TSR)**	**Power coefficient *C***_**p**_	**Tip speed ratio (TSR)**	**Power coefficient *C***_**p**_	**Tip speed ratio (TSR)**	**Power coefficient *C***_**p**_
0.02345	0.00427	0.04812	0.00656	0.04987	0.00887	0.04521	0.00739
0.03618	0.00691	0.05936	0.00911	0.05916	0.00915	0.04579	0.00751
0.03777	0.00692	0.06495	0.00921	0.06245	0.00967	0.06462	0.00838
0.04701	0.00706	0.06821	0.00956	0.0668	0.0126	0.06036	0.00854
0.04702	0.00739	0.0701	0.0133			0.07627	0.009972
0.053716	0.008499						

## 2 Experimental design, materials and methods

### 2.1 Wind turbine design and experimental setup

**Fig. 1:**
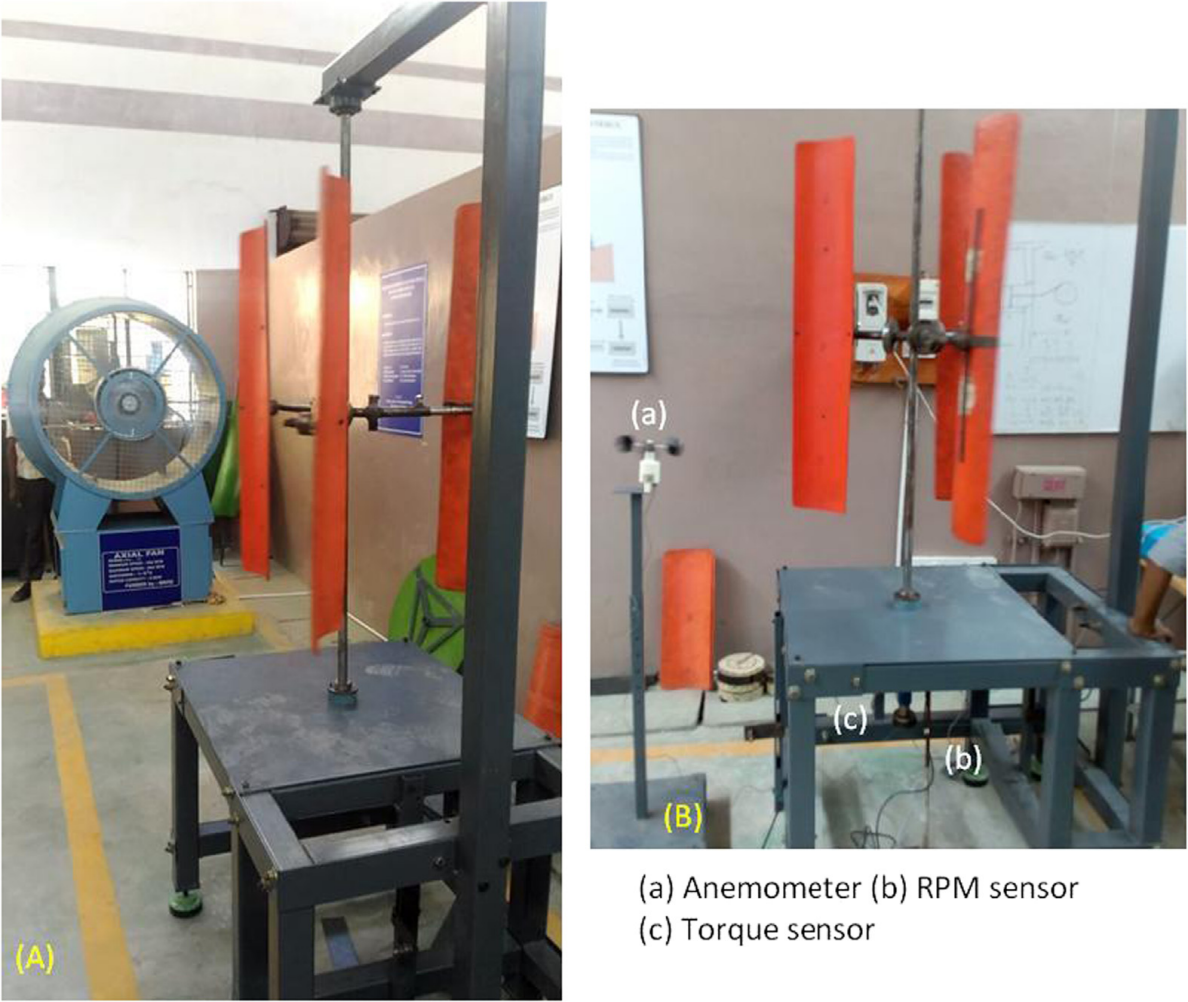
(a) Experimental setup (b) Close view of the proposed VAWT in test rig.

Fig. 1 highlights the experimental setup of the non-conventional H-rotor with semi-elliptic-shaped-bladed vertical axis wind turbine used for this data article. Cup type anemometer, torque sensor (Sushma™ make), non-contact type photo electric sensor is used to measure the wind velocity (*V*), torque in a rotating system (*T*) and rotating speed of the VAWT (*N*) which are recorded using NI^©^ data acquisition system with Labview^©^ software through a data logger interfaced with the computer. These raw data measured are processed and presented as a non-dimensional parameters namely, coefficient of power (*C*_p_), coefficient of torque (*C*_t_), tip speed ratio (*λ*) as represented by the wind turbine research community. The errors namely the random and bias of the data set acquired in this data are found as per the standard of error estimation [7] and the errors of the calculated parameters viz., the tip speed ratio and power coefficient is 2.0% and 3.0% respectively.

**Fig. 2:**
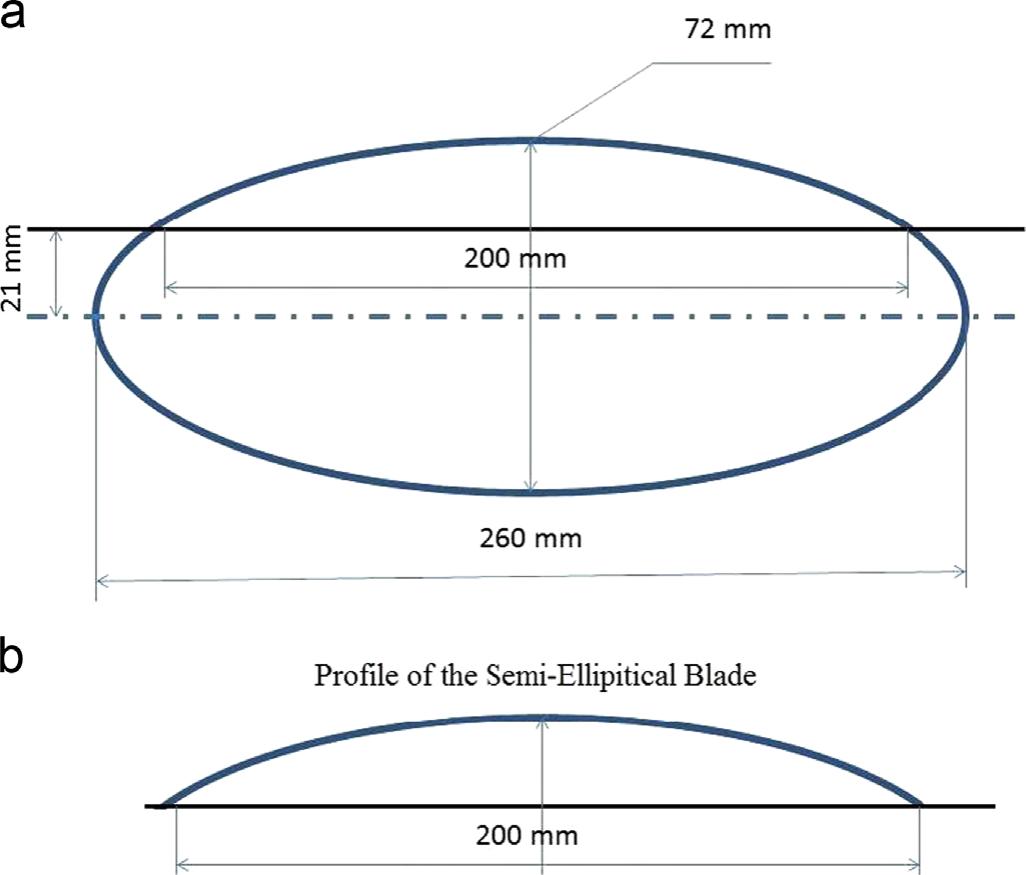
**a)** Dimensional details of the elliptical shape **b)** Cross-secitonal profile of the semi-elliptical blade.

The dimensional details and profile of the semi-elliptical blade is shown in Fig. 2. The specification detail of the proposed VAWT is given in Table 1. A brake drum type dynamometer is used to load the VAWT in order to measure the torque. One end of the 1 mm thick fishing nylon type wire string is fixed through a spring balance and the other end, which is connected to a weighing pan, is wound over the V-groove of the nylon drum attached to the rotor shaft for manual loading. Necessary precautions like reducing the friction of the bearing by washing it with petrol to remove the presence of grease and cooling the nylon drum over which the wire string is wounded with coolant water droplets in order to ensure proper loading of the rotor shaft are taken. The experiments to obtain this data set are carried out at Department of Mechanical Engineering, Hindustan Institute of Technology and Science, Padur, near Chennai. The velocity of the wind in this data is varied from 3 m/s to 6 m/s using an axial fan by a variable frequency drive (ABB^™^ make).

The non-conventional H-rotor VAWT consists of three blades which are positioned at 120° apart. Generally, three bladed arrangements are used for a wind turbine rotor as it has better self-starting characteristics along with proper balancing at low-wind speeds. The blades are made up of fibre reinforced plastics (FRP) material. An indexing mechanism with a ball and socket arrangement is used to tilt the blades by certain angles. In this data, the blades are tilted by 15° in the clockwise direction. The aspect ratio of the wind turbine rotor (As = H/D) is varied from 1.0 to 1.22 by varying the arm length to which the blades are attached.

In this data, the height of the rotor (*H*) is kept constant as 1 m and the diameter ‘*D*’ is estimated. The parameter ‘*D*’ is estimated by the expression given by

**Fig. 3:**
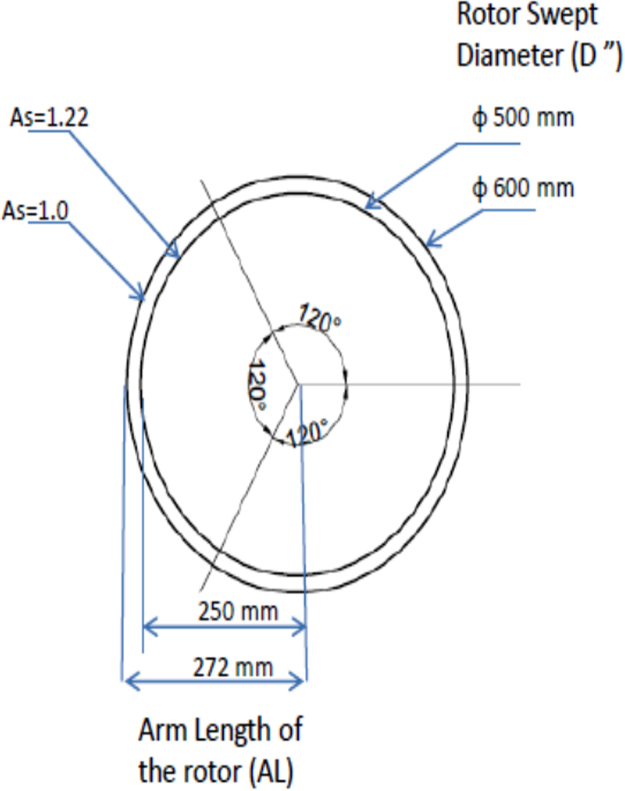
Arm length and projected diameter of rotor for different aspect ratios.

(1) D=2(d)+shd+AL

where D is the projected diameter of the rotor (*m*), d is the chord diameter of the blade (*m*), shd is the shaft diameter (m) and AL is the arm length of the rotor blade (*m*). Based on the aspect ratios, the projected diameters of the rotor are estimated. With the given chord diameter of the blades and shaft diameter which are fixed design parameters, the arm length of the rotor blade are estimated as shown in Fig. 3.

The performance parameters of this VAWT are reported in terms of coefficient of power and tip speed ratio. The tip speed ratio is given as

(2) $$\mathrm{Tip\backslash speed\backslash ratio}(\mathrm{TSR})\;=\;\frac{\mathrm{Velocity}\;\mathrm{of}\;\mathrm{the}\;\mathrm{turbine}\;\mathrm{rotor}}{\mathrm{Velocity}\;\mathrm{of}\;\mathrm{the}\;\mathrm{wind}}\;=\;\frac{2\pi \mathrm{rN}/60}{V}$$

where N is rotational speed (rpm) and *V* is the wind velocity (m/s). A given mass rate of free stream air (*ρAV*) flows over the swept area (*A*) and the power available in the air is given by the expression as

(3) $$\mathrm{Power\backslash Available}(P_{\mathrm{available}})\;=\;\frac{1}{2}\times \rho \times A\times V^{3}$$

The rotor shaft power generated (*P*_m_) is given by the expression as

(4) $$P_{m}\;=\;T\Omega \;=\;T\times \frac{2\pi N}{60}\;=\;\mathrm{Load\backslash applied\backslash x\backslash radius\backslash of\backslash the\backslash pulley}(r_{p})\times \frac{2\pi N}{60}$$

where ρ is density of air (kg/m^3^), *A* is swept area of the rotor (m^2^), *V* is velocity of the air (m/s), T is torque in N m and *N* is rotational speed of the rotor in RPM. The power coefficient (*C*_p_) is expressed as *C*_p_ = *P*_m_/*P*_available_

### 2.2 Performance and Loading test data

**Fig. 4:**
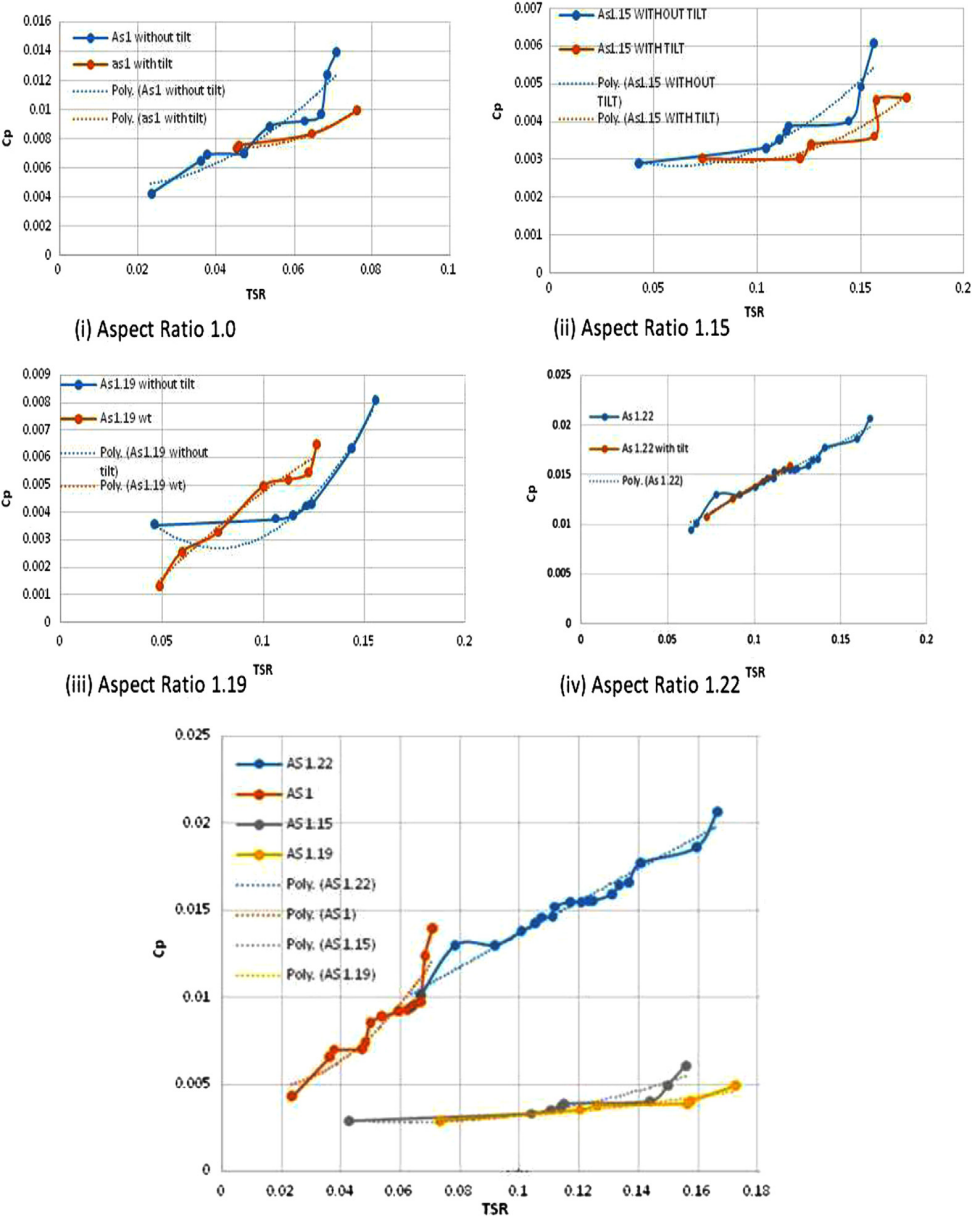
Variations of power coefficient (*C*_p_) with tip speed ratio (TSR) for various aspect ratios.

During experimentation, it is observed that the VAWT has the self-starting characteristics and it tend to rotate even at low-wind speed regimes. Table 2 shows the rotational speed of this proposed VAWT for various wind speed regimes. Fig. 4 shows the variations of power coefficient (*C*_p_) with tip speed ratio (TSR) for various aspect ratios (As) considered in this data. The blades without any tilt and blades with tilted conditions are tested for a wind velocity ranging from 3 m/s to 6 m/s. Table 3 shows the variations of TSR and *C*_p_ for an aspect ratio 1.22 for blades with and without tilt. As can be seen in Table 3, as the wind speed varies the turbine rotational speed also varies at constant loading condition. As can be seen in the Fig. 4 and Table 3, the coefficient of power reached the maximum value for an aspect ratio of 1.22. The power coefficient has reached its maximum value of 0.0207 with a TSR value of 0.1667 at a wind velocity of 5.54 m/s. Regarding aspect ratio 1.0 as seen in Table 4, it is clear that for 200 gm load, the coefficient of power is the maximum in comparison with other loads. As observed in Table 3, Table 4, the coefficient of power increases as the aspect ratio is increased for all loading conditions.

The study revealed that as the blade is tilted from 0 to 15 degrees in the clockwise direction, the performance of these VAWT with tilted blades in low-wind speed regimes is poor and not satisfactory at loads above 100 g and blades of this VAWT tend to stop rotating. In general, as the tip speed ratio increases, the power coefficient value also increases.

**Fig. 5:**
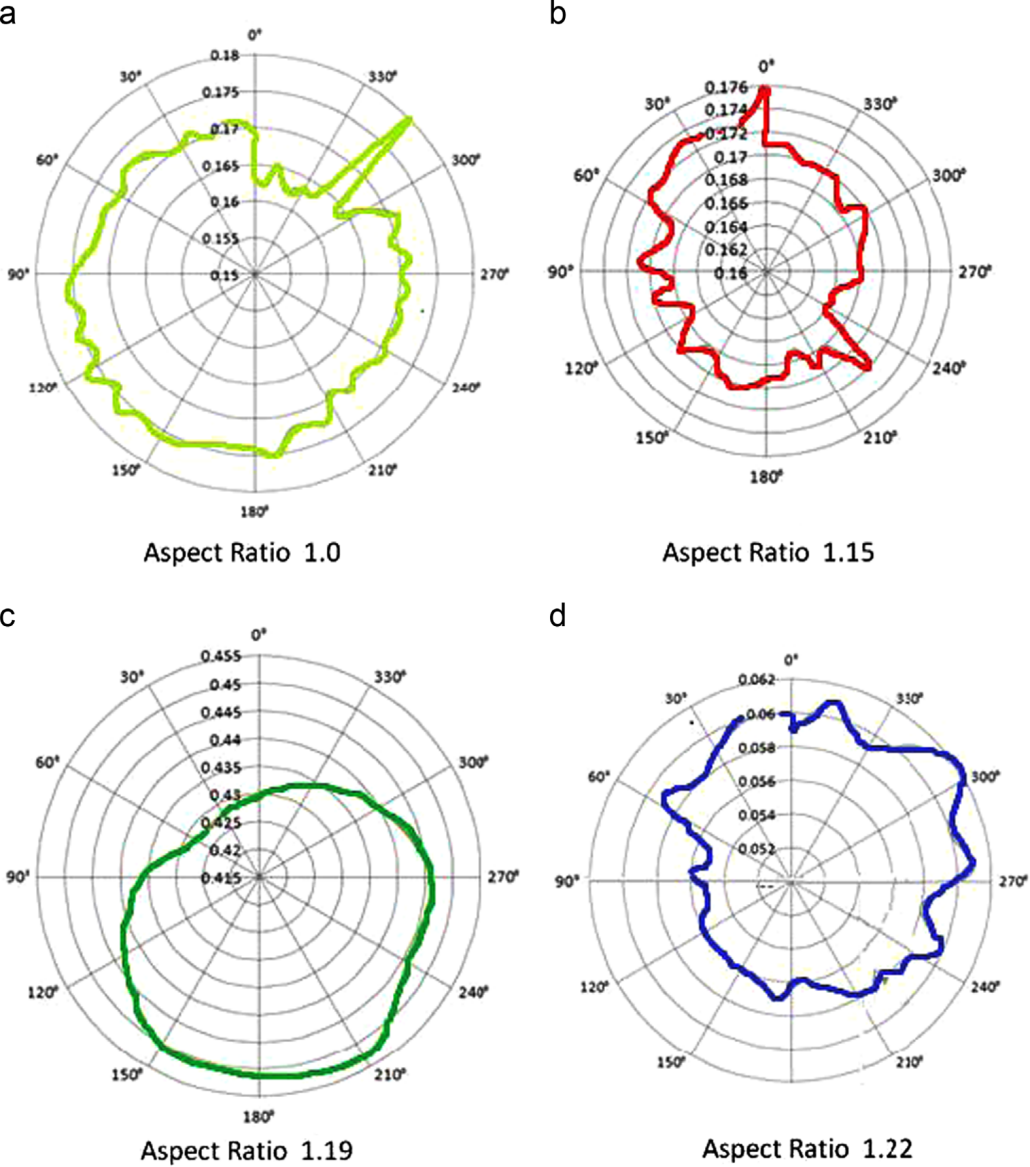
Comparison of the variations of torque with angle for different aspect ratios.

The polar plots for angle Vs torque for various aspect ratios is also plotted and studied in order to understand the distribution of torque coefficient at various angles of the blade in its 360 degree rotation. Fig. 5 shows the variations of torque with respect to angle in its rotational direction for an aspect ratio varying from 1.0 to 1.22. As can be seen from the polar chart Fig. 5(a), the torque gradually increases from 0° up to 180° and then it falls gradually in the remaining half of the revolution. A peak torque coefficient of 0.18 is recorded at 319° for this configuration. A peak torque coefficient of 0.173 is observed at 27 degrees of rotation of the VAWT for an aspect ratio of 1.15 (Fig. 5(b)). Similarly, a peak torque coefficient of 0.453 is observed at 210 degrees of rotation of the VAWT (Fig. 5(c)) and peak torque coefficient value of 0.062 at 307° of rotation (Fig. 5(d)) for an aspect ratio of 1.19 and 1.22 respectively. Except for aspect ratio 1.19, the torque coefficient is found to fluctuate for all the other aspect ratios. This may be due to the size of the blades of this type of VAWT kept in a free stream of air at certain wind velocities. Based on these polar charts, a general observation can be made that the peak values of torque are produced during the wind striking the advancing blade of the vertical axis wind turbine.
