# Supplementary material for: Experimental data of the study on H-rotor with semi-elliptic shaped bladed vertical axis wind turbine
Source: Data Brief. 2018 Jun 26;19:1828–36. doi: 10.1016/j.dib.2018.06.063 (PMC6141365; doi:10.1016/j.dib.2018.06.063)
Supplement: Supplementary file 1 — Transparency document [file mmc1.docx]

**Disclosure statement**

The authors state that there is no potential conflict of interest in the reported data.
